# COVID-19 induces more pronounced extracellular matrix deposition than other causes of ARDS

**DOI:** 10.1186/s12931-023-02555-7

**Published:** 2023-11-14

**Authors:** Natália de Souza Xavier Costa, Gabriel Ribeiro Júnior, Ellen Caroline Toledo do Nascimento, Jôse Mara de Brito, Leila Antonangelo, Caroline Silvério Faria, Jhonatas Sirino Monteiro, João Carlos Setubal, João Renato Rebello Pinho, Roberta Verciano Pereira, Marilia Seelaender, Gabriela Salim de Castro, Joanna D. C. C. Lima, Renata Aparecida de Almeida Monteiro, Amaro Nunes Duarte-Neto, Paulo Hilário Nascimento Saldiva, Luiz Fernando Ferraz da Silva, Marisa Dolhnikoff, Thais Mauad

**Affiliations:** 1 Departamento de Patologia (LIM 05), Faculdade de Medicina da Universidade de São Paulo, São Paulo, Brazil; 2 Laboratório de Investigação Médica (LIM03), Faculdade de Medicina, Hospital das Clínicas HCFMUSP, Universidade de São Paulo, São Paulo, Brazil; 3 Divisão de Patologia Clínica, Departamento de Patologia, Hospital das Clínicas HCFMUSP, Faculdade de Medicina, Universidade de São Paulo, São Paulo, Brazil; 4 Centro de Biologia Marinha, Universidade de São Paulo, São Sebastião, Brazil; 5 Departamento de Bioquímica, Instituto de Química Universidade de São Paulo, São Paulo, Brazil; 6 Cancer Metabolism Research Group, University of São Paulo, São Paulo, Brazil; 7 Department of Surgery and LIM 26, Hospital das Clínicas, University of São Paulo, São Paulo, Brazil; 8 Serviço de Verificação de Óbitos da Capital, Universidade de São Paulo, São Paulo, Brazil; 9 Departamento de Patologia, Laboratório de Patologia Ambiental (LIM- 05), Faculdade de Medicina da Universidade de São Paulo, Av. Dr. Arnaldo, 455, sala 1155, Cerqueira Cesar, São Paulo, Brazil

**Keywords:** COVID-19, Lung fibrosis, Extracellular matrix, Autopsy

## Abstract

**Background:**

Lung fibrosis is a major concern in severe COVID-19 patients undergoing mechanical ventilation (MV). Lung fibrosis frequency in post-COVID syndrome is highly variable and even if the risk is proportionally small, many patients could be affected. However, there is still no data on lung extracellular matrix (ECM) composition in severe COVID-19 and whether it is different from other aetiologies of ARDS.

**Methods:**

We have quantified different ECM elements and TGF-β expression in lung tissue of 28 fatal COVID-19 cases and compared to 27 patients that died of other causes of ARDS, divided according to MV duration (up to six days or seven days or more). In COVID-19 cases, ECM elements were correlated with lung transcriptomics and cytokines profile.

**Results:**

We observed that COVID-19 cases presented significant increased deposition of collagen, fibronectin, versican, and TGF-β, and decreased decorin density when compared to non-COVID-19 cases of similar MV duration. TGF-β was precociously increased in COVID-19 patients with MV duration up to six days. Lung collagen was higher in women with COVID-19, with a transition of upregulated genes related to fibrillogenesis to collagen production and ECM disassembly along the MV course.

**Conclusions:**

Fatal COVID-19 is associated with an early TGF-β expression lung environment after the MV onset, followed by a disordered ECM assembly. This uncontrolled process resulted in a prominent collagen deposition when compared to other causes of ARDS. Our data provides pathological substrates to better understand the high prevalence of pulmonary abnormalities in patients surviving COVID-19.

**Supplementary Information:**

The online version contains supplementary material available at 10.1186/s12931-023-02555-7.

## Introduction

The main cause of death in severe cases of COVID-19 is respiratory failure due to diffuse alveolar damage (DAD), the histological hallmark of acute respiratory distress syndrome (ARDS) [1].

The extension of pulmonary fibroproliferation in patients with early ARDS of different causes predicts increased mortality, ventilator dependency, and susceptibility to multiple organ failure [2]. The mortality rate is higher in patients with increased levels of TGF-β and procollagen type III in lung lavage fluids [3]. The pathological fibroproliferation associated with the lack of reabsorption of the provisional extracellular matrix (ECM), decreased fibrin clearance, matrix metalloproteinases (MMP), and tissue inhibitor of metalloproteinase (TIMP) imbalance, lead to residual fibrosis with pulmonary dysfunction in ARDS survivors [4]. Patients who die of ARDS show pulmonary fibrosis, even when they die in relatively early stages [3].

In severe COVID-19, the development of lung fibrosis is also a major concern. Wendish et al. [5] showed that SARS-CoV-2 triggered profibrotic macrophage responses and pronounced fibroproliferative ARDS. The profibrotic cytokine TGF-β seems to be involved in the early development of the disease, regulating the immune response, antibody production, and tissue remodelling [6]. An autopsy study conducted by Li et al. [7], found that 43% of fatal cases of COVID-19 showed fibrosing DAD.

Lung fibrosis in survivors of COVID-19 has a highly variable prevalence. A study from the United Kingdom showed that 7% of the patients presented lung fibrosis four months after hospital discharge [8], whereas a Chinese study showed that 60% of the patients presented lung fibrosis 3–5 months after the disease onset [1]. Given that millions of people were infected (and re-infected) with SARS-CoV-2, even if the long-term risk is proportionally small, there still could be a significant number of patients developing lung fibrosis.

The ECM has a broad range of functions, from the cellular scaffold to cell adhesion, migration, and survival being involved in several important biological processes. In the lungs, the ECM composition is especially important to its homeostasis, injury-repair response, and mechanical properties [9]. Collagen, elastic fibres, fibronectin and proteoglycans are major components of the ECM in the lungs, having different roles in maintaining structural and cell homeostasis [10]. In our previous studies, we have observed an increased fibroblast proliferation [11] and an early predominance of intermediate DAD, with loose aggregates of fibroblasts admixed with scattered inflammatory cells, and collagen deposition shortly after the onset of MV [12] in the lungs of patients with fatal COVID-19. However, there is still no data on ECM composition of lung remodelling in severe COVID-19 and whether it is different from other aetiologies of ARDS.

Therefore, in this study, we have analysed different ECM elements and TGF-β expression in lung tissue of fatal COVID-19 cases compared to other causes of ARDS. Furthermore, we analysed the transcriptomics and cytokines profile, in COVID-19 cases.

## Materials and methods

### Ethics

The National Research Ethics Commission (CONEP) approved this study (CAAE #30364720.0.0000.0068). Autopsies were performed after written informed consent by the next-of-kin.

### Study Population

We included all the autopsies of adult individuals performed between March and May of 2020 due to COVID-19 in Brazil (n = 28) when no vaccines were available. All COVID-19 patients had confirmation of SARS-CoV-2 infection by a positive RT-PCR result on the naso/oropharyngeal swab and/or lung tissue. During this period, there were 1898 admissions due to COVID-19 in the hospitals linked to the Medical School, with 659 deaths.

From May 2020 until July 2021, all autopsies were suspended in Brazil, and we had the approval to perform minimally invasive autopsy (MIA) for research purposes. MIA/US protocol in COVID-19 was described by Duarte-Neto et al. [13]. Lung tissues were collected during the MIA and the post-mortem interval ranged from 3 to 23 h and 51 min (mean of 22 h and 23 min). Lung tissue was sampled from eight regions, in a combination of the upper and lower chest (lateral and medial, 4 sites) and right and left lungs (2 sides). In each sampling site, six samples were collected. Immediately after sampling, pulmonary samples either were immersed in 10% formalin solution for 24 h or immediately snap frozen at -80 °C. For the immunohistochemistry analysis, two representative formalin-fixed paraffin-embedded biopsies (3–6 samples per slide) presenting DAD pattern and no histological signs of secondary bacterial/fungus infection were selected.

As controls, we selected retrospectively post-mortem lung tissue of 27 cases with criteria for non-COVID-19 ARDS diagnosis. Clinical diagnosis of ARDS was defined according to the Berlin definition [14] in addition to histological findings of diffuse alveolar damage (DAD) and the absence of chronic lung diseases. The cause of non-COVID-19 DAD in these patients was, in most of them, pulmonary or extrapulmonary sepsis. Lung tissues were collected during the conventional autopsy between 2004 and 2010. The post-mortem interval ranged from 4 h to 50 min to 21 h and 50 min (mean of 11 h and 18 min).

All patients were submitted to MV and both groups were further divided by MV duration: up to 6 days (L7) and 7 days or more (H7).

### xMAP Luminex Cytokine Multiplex Assay of COVID-19 cases

Samples of frozen lung tissue from one post-mortem biopsy site per case of the COVID-19 patients were prepared using Bio-plex Cell Lysis Kit (Bio-rad Laboratories, Hercules, CA, USA), following the manufacturer’s instructions. There was no availability of frozen tissue from the non-COVID-19 cases. Cytokines and chemokines were assessed using the following kits: Bio-plex TGF-β, Bio-plex Human Chemokine (Bio-rad, Hercules, California), and Milliplex MAP Human Cytokine/Chemokine (Millipore Corp., Billerica, MA) according to the kit-specific protocols. Measurements were made using a Magpix analytical test instrument by Luminex (Austin, TX). XPONENT 4.2 software and Milliplex Analyst 5.1 software (Millipore Corp., Billerica, MA, USA) were used for data analysis. To standardize the results, the total proteins of the lung samples were quantified with the TP2 kit (Roche Diagnostics GmbH, Mannheim, Germany) using COBAS C11 equipment (Roche Instrument Center, Rotkreuz, Switzerland). Data are expressed in pg/g and were correlated with clinical and histological parameters.

### Extracellular matrix analysis

Lung tissue slides were stained with Sirius Red (collagen), and Weigert’s resorcin-fuchsin with oxidation (elastic fibres). Lung tissue was immunostained using anti-Fibronectin, anti-Versican, anti-Decorin, and anti-TGF-β antibodies. Table S1 (Additional file 1) shows the commercial sources and dilutions of the antibodies. The ECM elements were quantified in the lung parenchyma, excluding airways, large blood vessels (only capillaries were not excluded), and pleura. The proportional area, positive stained area per lung parenchyma area (µm²/µm²) were measured in 15 high-power fields using the Image-Pro Plus 4.1 software (Media Cybernetics, Silver Spring, MD, USA). We selected a range of colors encompassing the positive staining for each marker, avoiding any background in the slide. There was no statistical difference in the analysed lung tissue area of each marker between the COVID-19 and non-COVID-19 groups (data not shown).

### Transcriptomics

For differential expression analysis, twelve COVID-19 cases were divided into 2 groups according to the MV duration: L7 (n = 5) and H7 (n = 7). We included control cases (n = 4) of individuals that died due to non-pulmonary causes. Characteristics of the control individuals are presented in Table S2 (Additional file 1). Data have been deposited in NCBI’s Gene Expression Omnibus (GSE205099).

Details about RNA extraction and sequencing are presented in supplementary material (Additional file 1). Data, read filtering and gene expression estimation were done as described in Erjefält et al. [11]. A preliminary analysis detected 23 genes related to heart tissue in two samples (Additional file 2 - Table S3) that were removed from further analysis. Heart tissue might contaminate lung biopsy during MIAS puncture trajectory. Due to the small amount of tissue recovery, the frozen sections were submitted to RNA extraction, without histological analysis. DESeq2 [15] was used to identify the differentially expressed genes (DEGs) between (1) L7 vs. control; (2) H7 vs. control; and (3) L7 vs. H7. For further details on the DEGs analysis, see the supplementary material (Additional file 1).

### Real-time PCR

Total RNA was extracted using Trizol (Thermo Fisher Scientific, Waltham, MA, USA) followed by a DNAse treatment (Thermo Fisher Scientific, Waltham, MA, USA) according to the manufacturer’s protocol. Reverse-transcribed cDNA samples were synthesized using High-Capacity cDNA Reverse Transcription Kit (Thermo Fisher Scientific, Waltham, MA, USA) also according to the manufacturer’s instructions. Real-time PCR reactions were conducted using QuantStudio 12 Instrument K Flex Real-Time PCR System (Applied Biosystems, Waltham, MA, USA) in the presence of Fast SYBR Green Master Mix (Thermo Fisher Scientific, Vilnius, Lithuania) and specific primers for the Collagen type I alpha 1 chain (*COL1A1*). The relative quantification was calculated after normalization against the levels of the reference gene ribosomal protein S18 (*RPS18*). Primer sequences are described in Table S4 (Additional file 1).

### Statistical analysis

SPSS 21 software (SPSS Inc/IBM Chicago, USA) was used for the statistical analyses. Data distribution was assessed by Shapiro-Wilk normality test. Depending on the data distribution, T-student or Mann-Whitney test were used to compare two groups and Kruskal–Wallis or one-way analysis of variance (ANOVA) test, followed by the Bonferroni or Tukey posthoc test to compare four groups. Statistical difference was assumed at the 5% significance level.

Factorial analysis of covariance (ANCOVA) was performed to explore influence factors on each dependent variable. We included COVID-19 and sex as factors and age, MV duration, BMI, and PaO_2_/FiO_2_ ratio as covariates. The covariates were added as main effects only and no interaction terms involving covariates were included. In addition, we calculated the partial eta squared (η^2^) as measures of effect size. Furthermore, we performed the Spearman correlation test between variables; coefficients (r) were considered statistically significant at p < 0.05.

## Results

### Demographics and clinical features

Lung samples from 28 COVID-19 patients (11 F/17 M), mean age of 56.9 years (range 32–88), were selected for this study. Part of this population has been previously presented [11, 12]. Table 1 and S5 (Additional file 1) show COVID-19 patients’ demographics, comorbidities, and clinical characteristics. There were 10 patients in the L7 group and 18 in the H7 group (demographics are shown in Table S7 - Additional file 1).

**Table 1:** Demographic and clinical characteristics of COVID-19 and non-COVID-19 patients

	COVID-19 (n = 28)	non-COVID-19 (n = 27)	p-value
**Age in years**, mean ± SD	56.9 ± 16.4	44.6 ± 14.7	0.005
**Body Mass Index (Kg/m**^**2**^**)**, mean ± SD	26.6 ± 7.8	24.4 ± 2.9	0.175
**Sex**, n (%)			
Male	17 (60.7%)	12 (44.4%)	0.227
Female	11 (39.3%)	15 (55.6%)	
**Race (self-declared)**, n (%)			
White	24 (85.7%)	18 (66.7%)	0.096
Afro-descendent	4 (14.3%)	9 (33.3%)	
**Comorbidities**, n (%)			
SAH	15 (53.6%)	7 (25.9%)	
Diabetes mellitus	10 (35.7%)	3 (11.1%)	
Cardiopathy	9 (32.1%)	3 (11.1%)	
Obesity	5 (17.8%)	2 (7.4%)	
COPD	4 (14.3%)	0	
Chronic renal disease	4 (14.3%)	1 (3.7%)	
Immunosuppression	4 (14.3%)	5 (18.5%)	
Vascular disease	4 (14.3%)	2 (7.4%)	
Neoplasia	3 (10.7%)	4 (14.8%)	
Asthma	2 (7.1%)	0	
HIV	0	5 (18.5%)	
Hepatic disease	0	7 (25.9%)	
No relevant comorbidities	5 (17.9%)	0	
**Smoking**, n (%)			
Yes	2 (7.1%)	4 (14.8%)	0.480
Former	7 (25%)	4 (14.8%)	
**Time from symptom onset to the hospitalization in days**, mean ± SD	5.5 ± 3.5		
**Time from symptom onset to death in days**, mean ± SD	18.7 ± 8.7		
**Period of hospitalization in days**, mean ± SD	12.5 ± 7.5	18.2 ± 13.7	0.121
**Mechanical ventilation duration in days**, mean ± SD	9.2 ± 6.5	7.1 ± 7.8	0.109
**PaO**_**2**_**/FiO**_**2**_**ratio**, mean ± SD	155 ± 95.7	163.7 ± 113.7	0.973

SAH, Systemic Arterial Hypertension; COPD, Chronic Obstructive Pulmonary Disease

The 27 non-COVID-19 DAD patients (15 F/12 M), mean age of 44.6 years (range 18–81), had non-pulmonary (n = 14) or pulmonary ARDS (n = 13). The most common underlying conditions for respiratory failure were bronchopneumonia (n = 14), followed by liver diseases (n = 11), extrapulmonary sepsis (n = 9), gastrointestinal diseases (n = 7) and bleeding (n = 5), cardiovascular diseases (n = 5), neurologic diseases (n = 5), and renal diseases (n = 4). Table 1 and S6 (Additional file 1) show non-COVID-19 patients’ demographics, comorbidities, and clinical characteristics. There were 17 patients in the L7 group and 10 in the H7 group (demographics are shown in Table S7 - Additional file 1).

There was no statistical difference in the MV and hospitalization duration between the COVID-19 and non-COVID-19 groups.

### ECM composition: COVID-19 DAD x non-COVID-19 DAD

**Fig. 1:**
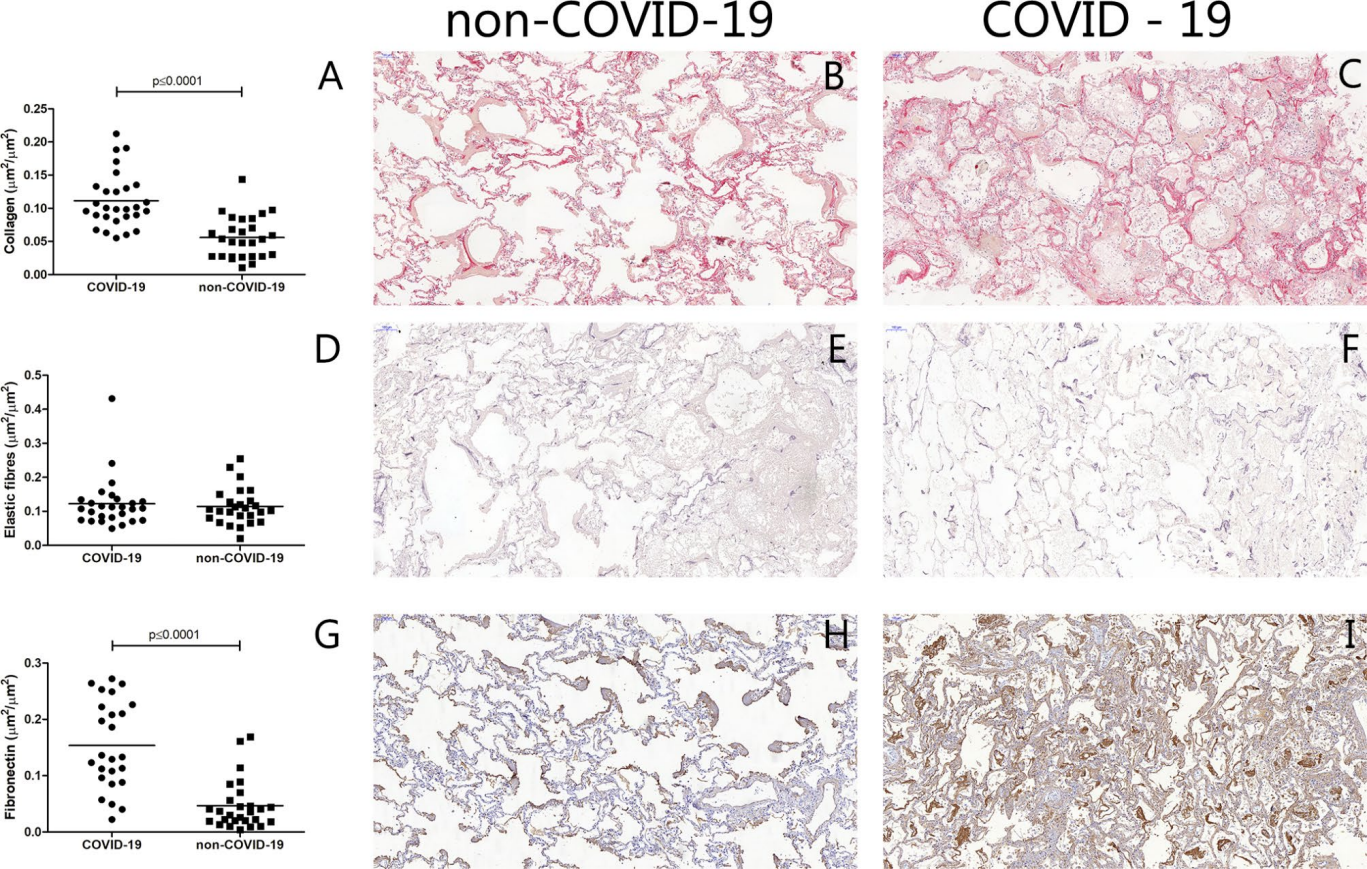
Graphical representation of the lung tissue extracellular matrix composition: Comparison between the COVID-19 and non-COVID-19 cases. **A**: Collagen (µm²/ µm²). Photomicrographs of non-COVID-19 (**B**) and COVID-19 (**C**) stained with Sirius red. **D**: Elastic Fibres (µm²/ µm²). Photomicrographs of non-COVID-19 (**E**) and COVID-19 (**F**) stained with Weigert’s resorcin-fuchsin with oxidation. **G**: Fibronectin (µm²/ µm²). Photomicrographs of non-COVID-19 (**H**) and COVID-19 (**I**) immunostainned with anti-fibronectin antibody. Bars show the mean and each point represents one patient. Scale bar = 100 μm

**Fig. 2:**
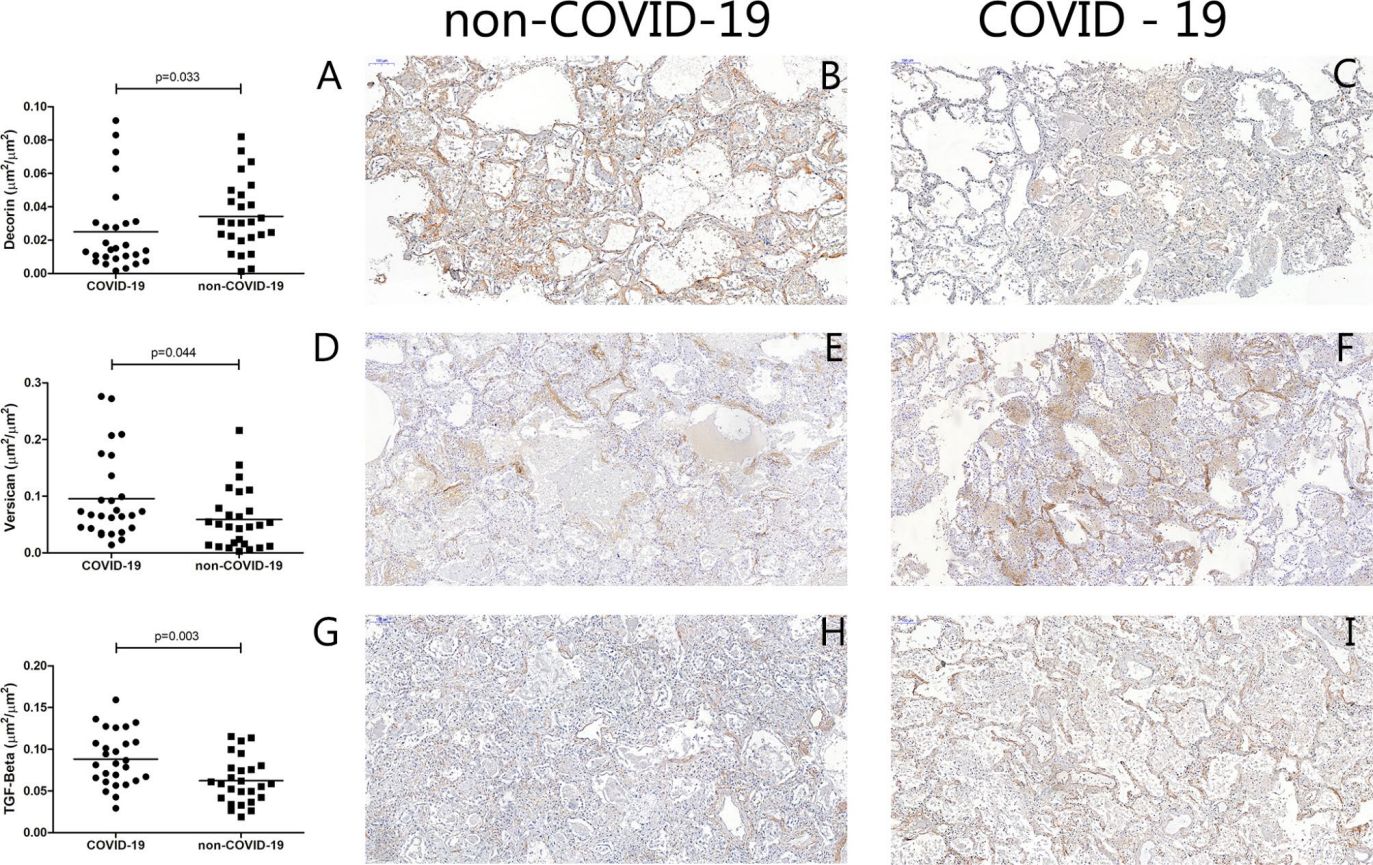
Graphical representation of the lung tissue extracellular matrix composition: Comparison between the COVID-19 and non-COVID-19 cases. **A**: Decorin (µm²/ µm²). Photomicrographs of non-COVID-19 (**B**) and COVID-19 (**C**) immunostainned with anti-decorin antibody. **D**: Versican (µm²/ µm²). Photomicrographs of non-COVID-19 (**E**) and COVID-19 (**F**) immunostainned with anti-versican antibody. **G**: TGF-beta (µm²/ µm²). Photomicrographs of non-COVID-19 (**H**) and COVID-19 (I) immunostainned with anti-TGF-beta antibody. Bars show the mean and each point represents one patient. Scale bar = 100 μm

The collagen density was increased in the COVID-19 cases compared to the non-COVID-19 (p < 0.0001) (Fig. 1). There was no statistically significant difference in elastic fibre density between the groups (Fig. 1). The fibronectin (p < 0.0001) and the versican (p = 0.044) densities were increased in the COVID-19 cases compared to the non-COVID-19, whereas the decorin density was decreased in the COVID-19 cases compared to the non-COVID-19 (p = 0.033) (Figs. 1 and 2). In addition, the expression of TGF-β was higher in the COVID-19 than in the non-COVID-19 cases (p = 0.003) (Fig. 2).

### The influence of MV duration, age, BMI, and PaO _2_/FiO_2_ ratio on the ECM composition and TGF-β

Table 2 shows the results of the ANCOVA analysis.

**Table 2:** Factorial ANCOVA analysis

	COVID-19	Sex	Interaction*	MV duration	Age	BMI	PaO_2_/FiO_2_ ratio
Collagen							
F_(1,38)_	**23.7**	**9.24**	3.29	0.32	1.58	1.89	0.65
p-value	**< 0.0001**	**0.004**	0.077	0.572	0.216	0.177	0.425
partial η^2^	**0.385**	**0.196**	0.08	0.008	0.040	0.048	0.017
**Elastic Fibres**							
F_(1,37)_	1.25	1.88	0.18	**7.14**	0.09	1.30	1.263
p-value	0.271	0.179	0.677	**0.011**	0.758	0.261	0.268
partial η^2^	0.033	0.048	0.005	**0.162**	0.003	0.034	0.033
**Fibronectin**							
F_(1,36)_	**18.94**	0.822	0.69	0.387	0.475	0.50	0.759
p-value	**< 0.0001**	0.371	0.409	0.538	0.495	0.484	0.389
partial η^2^	**0.345**	0.022	0.019	0.011	0.013	0.014	0.021
**Versican**							
F_(1,37)_	**6.87**	2.61	0.007	0.10	0.001	1.99	0.947
p-value	**0.013**	0.114	0.935	0.754	0.981	0.167	0.337
partial η^2^	**0.157**	0.066	0	0.003	0	0.051	0.025
**TGF-β**							
F_(1,37)_	**6.09**	0.065	0.683	**4.37**	0.048	0.884	0.291
p-value	**0.018**	0.80	0.414	**0.044**	0.828	0.353	0.593
partial η^2^	**0.141**	0.002	0.018	**0.106**	0.001	0.023	0.008
**Decorin**							
F_(1,38)_	**5.25**	3.99	0.025	0.73	0.97	0.189	0.596
p-value	**0.028**	0.053	0.875	0.399	0.329	0.666	0.445
partial η^2^	**0.124**	0.097	0.001	0.019	0.026	0.005	0.016

* Interaction between the factors COVID-19 and sex. F (df, df error). Partial η^2^ = partial eta squared are measures of effect size; it represents the proportion of variance in the dependent variable associated with an effect. MV: Mechanical Ventilation. We observed no significant correlation among the covariates

All assessed ECM components, collagen (p < 0.0001; η^2^ = 0.38), fibronectin (p < 0.0001; η^2^ = 0.34), versican (p = 0.013; η^2^ = 0.15), decorin (p = 0.028; η^2^ = 0.12), and TGF-β (p = 0.018; η^2^ = 0.14), had their expression influenced by COVID-19, except the elastic fibres (p = 0.271; η^2^ = 0.03).

**Fig. 3:**
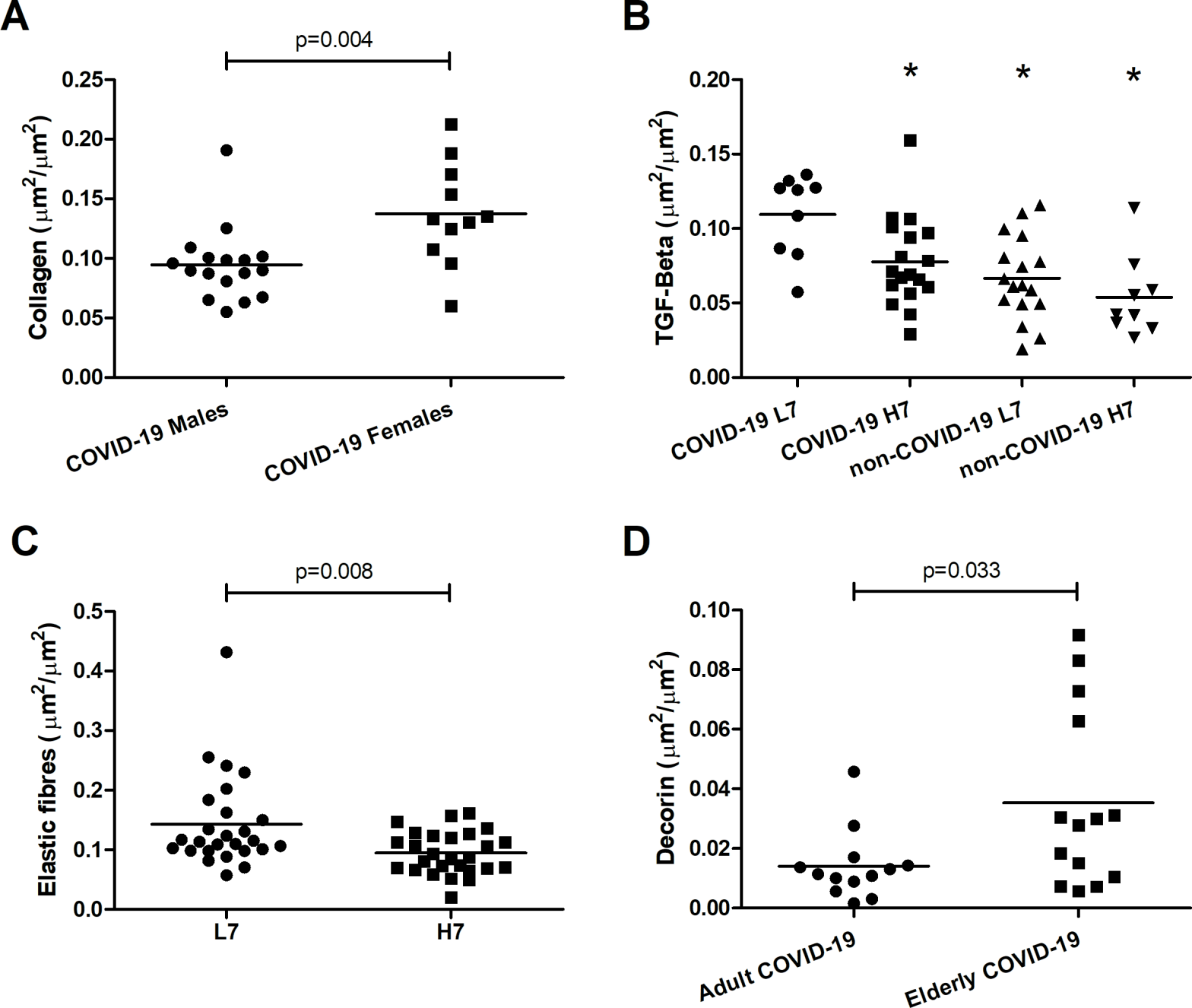
Graphical representation of the lung tissue extracellular matrix composition. **A**: Collagen density – COVID-19 Males x COVID-19 Females (µm²/ µm²). **B**: TGF-β expression divided by the duration of the mechanical ventilation (µm²/ µm²). **C**: Elastic fibre density divided by the duration of the mechanical ventilation (µm²/ µm²). **D**: Decorin density – Adult COVID-19 x Elderly COVID-19 (> 60 years of age) (µm²/ µm²). L7: up to 6 days of mechanical ventilation; H7: more than 7 days of mechanical ventilation. *p < 0.05 compared to the COVID-19 L7 group. Bars show the mean and each point represents one patient

The only component that was influenced by sex was collagen (p = 0.004; η^2^ = 0.19). The COVID-19 patients of the female sex presented higher density of collagen in the lung tissue (p = 0.004) (Fig. 3A). Between the COVID-19 patients of the female and male sex, we did not observe a difference in age, MV duration, BMI, or PaO_2_/FiO_2_ ratio. There was no difference in the collagen between males and females in the non-COVID-19 group. The characteristics of males and females are presented in Table S8 (Additional file 1).

The components influenced by the MV duration were the TGF-β (p = 0.044, η^2^ = 0.11) and elastic fibres (p = 0.011, η^2^ = 0.16). When the groups are divided by the MV duration, the COVID-19 L7 group had higher TGF-β density than the non-COVID-19 L7 and the non-COVID-19 H7 groups (p = 0.003 and p = 0.001, respectively). In addition, the COVID-19 L7 has higher density of TGF-β than the COVID-19 H7 group (p = 0.043) (Fig. 3B). Considering all patients, regardless of COVID-19, there was a decrease in elastic fibres in the H7 cases when compared to L7 (p = 0.008) (Fig. 3C).

There was a positive correlation between age and decorin density (r = 0.411; p = 0.022). Elderly COVID-19 cases (> 60 years of age) had higher decorin than the adult COVID-19 cases (p = 0.033) (Fig. 3D).

### Correlations between the ECM components, cytokines/chemokines, and clinical variables in COVID-19 cases

**Fig. 4:**
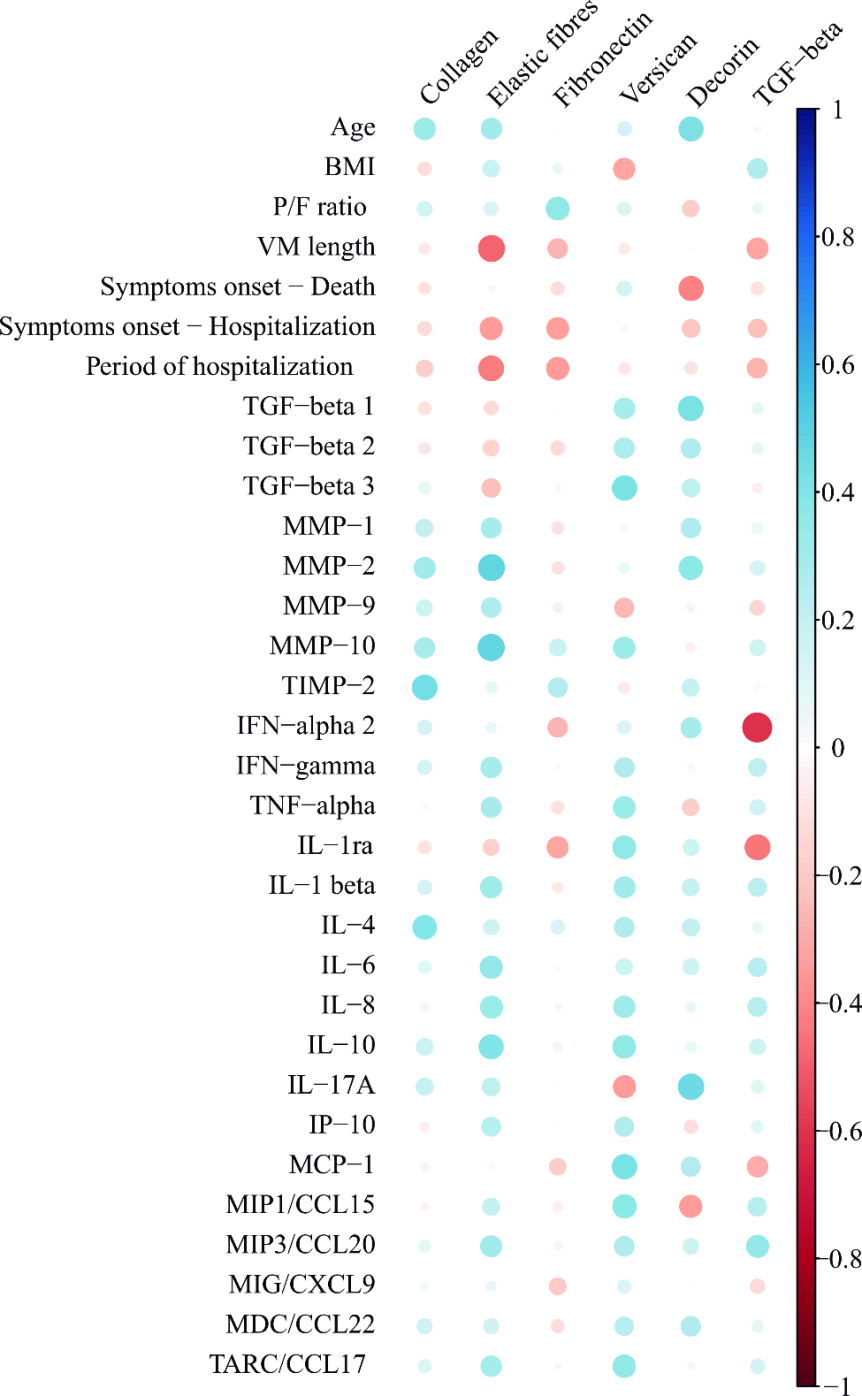
Correlation plot showing the correlations between histological analysis of the lung tissue extracellular matrix elements, TGF-beta protein expression, cytokines and clinical data of COVID-19 patients. The specific correlation coefficients and p-values of the statistically significant correlations are presented in the main text. BMI: body mass index. P/F ratio: PaO_2_/FiO_2_ ratio. MV: mechanical ventilation. Symptoms onset – Death: Time from symptom onset to death. Symptoms onset - Hospitalization: Time from symptom onset to hospitalization. TGF-beta: Transforming growth factor-beta. MMP: metalloproteinase. IFN: interferon. TNF: Tumor necrosis factor. IL: Interleukin. IP-10: Interferon-gamma inducible Protein 10 kDa. MCP: Monocyte chemoattractant protein. MIP: Macrophage inflammatory protein. CCL: Chemokine (C-C motif) ligand. MIG: Monokine induced by interferon-gamma. CXCL: chemokine (C-X-C motif) ligand. TARC: Thymus and activation-regulated chemokine

Data showing the mean concentration levels of the cytokines/chemokines are shown in Table S9 (Additional file 1). Figure 4 shows the correlation plot.

The collagen density was positively correlated with the elastic fibre density (r = 0.528; p = 0.005) and TIMP2 (r = 0.435; p = 0.034).

The elastic fibre density was positively correlated with the MMP2 (r = 0.483; p = 0.02) and MMP10 (r = 0.489; p = 0.011). Moreover, it was negatively correlated with the MV duration (r=-0.483; p = 0.011) and with the time elapsed between hospital admission and death (r=-0.439; p = 0.022).

Versican density was positively correlated with TGF-β3 (r = 0.423; p = 0.045), and the decorin density was positively correlated with the TGF-β1 levels (r = 0.428; p = 0.042) and age (r = 0.411; p = 0.022). Decorin was also negatively correlated with the interval between the onset of symptoms and hospital admission (r=-0.414; p = 0.032). TGF-β density was negatively correlated with IFN-α2 (r=-0.604, p = 0.022).

### Increased expression of collagen-related genes and fibrillogenesis in severe COVID-19 patients

When compared to the control cases, the COVID-19 L7 cases showed 70 DEGs (54 up- and 16 down-regulated) and the COVID-19 H7 showed 208 DEGs (139 up- and 69 down-regulated). The COVID-19 L7 compared to the COVID-19 H7 showed 46 DEGs (34 up- and 12 down-regulated).

DEG analysis revealed increased expression of genes related to collagen assembly and organization in the L7 group compared to the control, such as tolloid-like 2, ADAMTS8, and collagen III. Group L7 had up-regulated genes related to complement activation, humoral immune response and B cell activation, phagocytosis, activation of the immune response, and mitosis (Table S10 - Additional file 3).

The DEG analysis in the H7 group compared to the control showed increased expression of genes related to fibrillogenesis and ECM degradation, such as biglycan, collagen types I, III, and V, CTHRC1, MMP-8, MMP-9, and cathepsin G. The up-regulated genes were related to phagocytosis, complement activation, immune response, B cell activation and immunoglobulin production, mitosis, neutrophil-mediated immunity, collagen synthesis, ECM disassembly, and regulation of homotypic cell-cell adhesion (Table S11 - Additional file 4).

When we compared COVID-19 L7 to the COVID-19 H7 group, the downregulated genes were related to response to interleukin-1, leukocyte migration and neutrophil chemotaxis, humoral immune response, cell adhesion molecule production, and negative regulation of proteolysis (Table S12 - Additional file 5).

Although the gene expression of *COL1A1* assessed by real-time PCR was increased in the COVID-19 H7 (4.36 ± 2.9; n = 7) and compared to the control (1.01 ± 0.21; n = 3), we did not observe the statistical difference (p = 0.143), probably due to the low number of samples analysed.

## Discussion

In this study, we have shown that COVID-19 cases present increased deposition of collagen, fibronectin, versican, and TGF-β in the lungs when compared to non-COVID-19 ARDS cases of similar ventilation duration. ECM deposition occurred early after MV onset in both groups, and TGF-β was precociously increased in COVID-19. There was a transition of upregulated genes related to fibrillogenesis to collagen production and ECM disassembly along the MV course, with lung collagen was higher in women with COVID-19. To our knowledge, this is the first study to address the ECM composition and its influence factors in COVID-19.

Our data reassure the central role of TGF-β in the remodelling taking place in severe COVID-19. TGF-β can be secreted by different cell populations of lung inflammatory and structural cells [6]. Xu et al. [16] showed that TGF-β expression was increased in alveolar epithelial cells after SARS-CoV-2 infection, which could block apoptosis of the infected host cells, promoting tissue fibrosis [17]. Vaz de Paula et al. [18] showed increased lung TGF-β expression in fatal COVID-19 when compared to H1N1 patients.

There are some mechanisms to help explain the early and excessive TGF-β increase in fatal COVID-19 cases, as observed in this study. In severe cases, SARS-CoV-2 induces poor interferon production [19], which could explain the increased TGF-β expression, since IFN-γ specifically inhibits an early step of TGF-β-induced activation of Smad3 [20]. In our data, we also observed a negative correlation between TGF-β density and IFN-α2. Accordingly, Hu et al. [21] found a negative correlation between the lung fibrotic volume and two circulating interferons, IFN-gamma and IFN-α2, at the discharge of COVID-19 patients.

We observed a decrease in the decorin density in COVID-19 lungs. Decorin regulates fibrillogenesis acting as an anti-fibrotic agent, partially via binding and neutralizing the TGF-β [22] and it may also bind to collagen fibrils aiding in the stabilization of collagen [23]. We observed a positive correlation between decorin density and TGF-β1 levels. TGF-β1 can downregulate the decorin synthesis in fibroblasts and, in turn, decorin can inhibit TGF-β1 [24]. We also observed an increased biglycan gene expression in COVID-19 cases with more than 7 days of VM. Unlike decorin, biglycan may be responsible for the sequestration of TGF-β to cell-surface receptors, promoting ECM formation [25].

We observed an important increase in fibronectin in the COVID-19 cases. Xu et al. [16] showed that the SARS-CoV-2 infection increases the fibronectin gene expression in alveolar epithelial cells. In situations of lung remodelling, fibronectin is one of the first molecules to be deposited in the extracellular space, and it regulates assembly and stability of several ECM components [26]. Furthermore, the binding of fibronectin with latent TGF-β binding proteins and later activation by proteases and integrins are major regulators of TGF-β bioavailability and signalling [26]. Fibronectin levels were increased in the plasma of COVID-19 patients compared to non-COVID-19 patients [27] and even higher in fatal COVID-19 [28].

The versican density was also increased in COVID-19. Versican plays an important role in immunity and inflammation influencing key events such as cell adhesion, proliferation, migration, and ECM remodelling [29]. Versican mRNA can be differentially spliced generating five isoforms (V0-V4), which differently affect cell behaviour [29, 30]. We observed a positive correlation between versican and TGF-β3. TFG-β3 induces the expression of the variants V0 and V1 in fibroblasts [31], which may perpetuate tissue remodelling by increasing proliferation and resistance to apoptosis of fibroblasts [32]. Moreover, we observed the increased expression of several genes linked to mitosis including Ki-67, as per our previous study [11], indicating that COVID-19 drives the lungs to a highly fibroproliferative state.

There was an increased deposition of collagen in COVID-19 lungs, with an early increased expression of genes linked to ECM orchestration and organization, such as Tolloid-like 2, ADAMTS8, and collagen III. After seven days of VM, there was increased gene expression of collagen I, III, V, and CTHRC-1. Interestingly, a lung cell atlas study identified a subpopulation of CTHRC-1 + fibroblasts, present exclusively within fibrotic areas expressing collagen I, III, and V [33]. Increased gene expression of collagens was accompanied by the upregulation of MMP-8, MMP-9, and cathepsin G. In addition, there was a positive correlation between collagen density and TIMP-2 and a higher collagen density in females compared to males. Although sex hormones are likely to have some influence over fibrillogenesis, their role is poorly understood. Although it is not unanimous, many studies already showed that the female sex is associated with an increased risk for long COVID-19 [34–37].

The increased ECM deposition and protease expression represent a dynamic process of deposition and degradation that may lead to destabilization. Furthermore, endogenous molecules, such as versican and biglycan, can interact with toll-like receptors, inducing the production of proinflammatory cytokines [30]. Interestingly, we observed a negative correlation between the elastic fibres and VM duration, and a positive correlation with MMP-2 and MMP-10. It has been described that the products resulting from elastic fibre hydrolysis can induce leukocyte activation, inhibit elastase activity, and stimulates fibroblast proliferation [38].

Taken together, our data suggest that in severe cases of COVID-19, an early expression of TGF-β environment and ECM-rich synthesis and degradation takes place, leading to a more pronounced lung remodelling than other causes of ARDS. Our data complement the work of Mothes et al. [39], which showed the role of a non-resolving local immune response that extends beyond active viral infection and perpetuates tissue remodelling.

Our study has some limitations, such as the low number of the studied cases, mainly in the transcriptomic analysis and PCR, due to the difficulties in obtaining COVID-19 autopsy tissue during the pandemics, including ones with good RNA quality. Another limitation was the lack of frozen tissue from the non-COVID-19 cases which limits our interpretation regarding the differences between COVID-19 and non-COVID-19. We do not have cases with very prolonged ventilation periods, as not infrequently observed in some COVID-19 cases along the pandemic course, as these cases were collected very early during the pandemic in Brazil. Since all patients had a fatal outcome, we do not know how long ECM abnormalities in the lung would persist. A study performed on COVID-19 survivors hospitalized at our Medical School in Sao Paulo during the same period showed that 30% had persistent dyspnoea and 32% had reduced forced vital capacity [37].

In summary, our data show that severe COVID-19 is associated with an early expression of TGF-β lung environment after MV onset. This particular environment led to a disordered ECM assembly with upregulation of genes related to ECM fibrillogenesis and disassembly. This uncontrolled process resulted in a prominent collagen deposition when compared to other causes of ARDS. Our data provides pathological substrates to better understand the high prevalence of pulmonary abnormalities in patients surviving COVID-19.

### Electronic supplementary material

Below is the link to the electronic supplementary material.

Supplementary Material 1

Supplementary Material 2

Supplementary Material 3

Supplementary Material 4

Supplementary Material 5

## Data Availability

Data on transcriptome reads have been deposited in NCBI’s Gene Expression Omnibus and are accessible through GEO Series accession number GSE205099 (https://www.ncbi.nlm.nih.gov/geo/query/acc.cgi?acc=GSE205099). Further data are available from the corresponding author upon reasonable request.
