# Supplementary material for: COVID-19 induces more pronounced extracellular matrix deposition than other causes of ARDS
Source: Respir Res. 2023 Nov 14;24:281. doi: 10.1186/s12931-023-02555-7 (PMC10648646; doi:10.1186/s12931-023-02555-7)
Supplement: Supplementary file 1 — Supplementary Material 1 [file 12931_2023_2555_MOESM1_ESM.docx]

**Supplementary material**

**COVID-19 induces more pronounced extracellular matrix deposition than other causes of ARDS**

Natália de Souza Xavier Costa^1^, Gabriel Ribeiro Júnior^1^, Ellen Caroline Toledo do Nascimento^1^, Jôse Mara de Brito^1^, Leila Antonangelo^2,3^, Caroline Silvério Faria^2^, Jhonatas Sirino Monteiro^4^, João Carlos Setubal^5^, João Renato Rebello Pinho^2^, Roberta Verciano Pereira^2^, Marilia Seelaender^6,7^, Gabriela Salim de Castro^6,7^, Joanna D. C. C. Lima^6,7^, Renata Aparecida de Almeida Monteiro^1^, Amaro Nunes Duarte-Neto^1^, Paulo Hilário Nascimento Saldiva^1^, Luiz Fernando Ferraz da Silva^1,8^, Marisa Dolhnikoff^1^, Thais Mauad^1*^.

1 Departamento de Patologia, LIM-05, Faculdade de Medicina da Universidade de São Paulo, São Paulo, Brazil.

2 Laboratório de Investigação Médica (LIM03), Hospital das Clínicas HCFMUSP, Faculdade de Medicina, Universidade de São Paulo, São Paulo, Brazil

3 Divisão de Patologia Clínica – Departamento de Patologia, Hospital das Clínicas HCFMUSP, Faculdade de Medicina, Universidade de São Paulo, São Paulo, Brazil

4 Centro de Biologia Marinha, Universidade de São Paulo, São Sebastião, Brazil

5 Departamento de Bioquímica, Instituto de Química Universidade de São Paulo, São Paulo, Brazil

6 Cancer Metabolism Research Group, University of São Paulo, Brazil.

7 Department of Surgery and LIM 26, Hospital das Clínicas, University of São Paulo, São Paulo, Brazil.

8 Serviço de Verificação de Óbitos da Capital, Universidade de São Paulo, São Paulo, Brazil.

**Table of Contents**

[**Supplementary Methods** 4](#_Toc140580598)

[**Table S1** 4](#_Toc140580599)

[**Table S2** 4](#_Toc140580600)

[**RNA extraction and sequencing** 5](#_Toc140580601)

[**DEGs analysis** 5](#_Toc140580602)

[**Table S3** 6](#_Toc140580603)

[**Table S4** 6](#_Toc140580604)

[**Supplementary Results** 7](#_Toc140580605)

[**Table S5** 7](#_Toc140580606)

[**Table S6** 8](#_Toc140580607)

[**Table S7** 9](#_Toc140580608)

[**Table S8** 10](#_Toc140580609)

[**Table S9** 11](#_Toc140580611)

[**Table S10** 11](#_Toc140580612)

[**Table S11** 12](#_Toc140580613)

[**Table S12** 12](#_Toc140580614)

[**References** 12](#_Toc140580615)

# **Supplementary Methods**

## **Table S1**. Standardized dilutions of the antibodies and their commercial sources.

| Target | Vendor (SKU) | Concentration |
| --- | --- | --- |
| Decorin | Sigma-Aldrich, St. Louis, Missouri, USA (HPA003315) | 1:600 |
| Fibronectin | Agilent, Santa Clara, California, USA (A0245) | 1:7000 |
| TGF-beta | Santa Cruz Biotechnology, Heidelberg, DE (sc-130348) | 1:600 |
| Versican | Seikagaku Corporation, Tokyo, JP (270428-1) | 1:4000 |

## **Table S2.** Demographic characteristics of control patients of the transcriptomic analyses.

| **Control Cases** | **Sex** | **Age (years)** | **BMI** | **Relevant Comorbidities** |
| --- | --- | --- | --- | --- |
| C1 | Female | 78 | 27 | Systemic arterial hypertension  Chronic renal disease  Cardiomyopathy |
| C2 | Male | 70 | 24.1 | Diabetes mellitus  Systemic arterial hypertension |
| C3 | Female | 77 | 21.1 | Cardiomyopathy  Smoker |
| C4 | Female | 46 | 23.1 | Systemic arterial hypertension  Adrenal Cancer |

## **RNA extraction and sequencing**

RNA was extracted using RNeasy Mini Kit (Qiagen, Hilden, Germany) according to the manufacturer’s protocol followed by a DNAse treatment with Turbo DNAse Kit (Thermo Fisher Scientific, Waltham, MA, USA) at 37°C for 30 min. The concentration was determined using the Qubit® RNA HS Assay Kit (Thermo Fisher Scientific, Waltham, MA, USA), and the quality was analyzed using the Agilent 2200 TapeStation System with the RNA ScreenTape assay (Santa Clara, CA, USA). For RNA-sequencing (RNA-Seq), libraries were prepared using Illumina® RNA Prep with Enrichment kit, nt, IDT® for Illumina® DNA/RNA UD Indexes Set A, Tagmentation (Illumina, San Diego, CA, USA) and Illumina Exome Panel – Enrichment Oligos Only (Illumina, San Diego, CA, USA). Libraries were validated by quantification using Qubit dsDNA HS Assay kit (Invitrogen, Thermo Fisher Scientific, Waltham, MA, USA) and size measurement using Agilent 2200 TapeStation System with the High Sensitivity DNA ScreenTape assay. Sequencing was performed on the Illumina NovaSeq 6000 system to generate 150-bp paired-end reads.

## **DEGs analysis**

Data, read filtering, and gene expression estimation were done as described in Erjefält et al. [1] A preliminary analysis detected 23 genes related to heart tissue (Table S3) that were removed from further analysis. Heart tissue might contaminate lung biopsy during MIAS puncture trajectory. Due to the small amount of tissue recovery, these frozen sections were submitted to RNA extraction only, without histological analysis. DESeq2 [2] was used to identify the differentially expressed genes (DEGs) between (1) L7 vs. control; (2) H7 vs. control; and (3) L7 vs. H7. A gene was considered differentially expressed if FDR ≤ 0.05. ClusterProfiler [3,4], together with genome annotations from Genome-wide annotation for Human (org.Hs.eg.db) R package (v3.13.0), was used to verify the Gene Ontology (GO) terms that were enriched among the DEGs. GO terms were considered enriched if the adjusted p-value was less than or equal to 0.05 [5].

## **Table S3.** Removed genes related to the heart tissue.

## **Table S4.** Primer Sequences

| Gene | Symbol | Primer Sequence | |
| --- | --- | --- | --- |
| Collagen type I alpha 1 chain | *COL1A1* | Forward | 5’-AGAGGTTTCCCTGGCGA-3’ |
|  |  | Reverse | 5’-ACCAGCATCACCCTTAGCA-3’ |
| Ribosomal protein S18 | *RPS18* | Forward | 5’-CCTGCGGCTTAATTTGACTC-3’ |
|  |  | Reverse | 5’-ATGCCAGAGTCTCGTTCGTT-3’ |

# **Supplementary Results**

## **Table S5.** Initial symptoms presented by COVID-19 patients.

| **Initial Symptoms*,** n (%) | **COVID-19 patients (n=28)** |
| --- | --- |
| Dyspnea | 23 (82.14%) |
| Fever | 19 (67.8%) |
| Cough | 18 (64.3%) |
| Myalgia | 8 (28.6%) |
| Nausea/Vomiting | 8 (28.6%) |
| Diarrhea | 4 (14.3%) |
| Rhinorrhea | 4 (14.3%) |
| Sore Throat | 3 (10.7%) |

*Some patients may have more than one initial symptom.

## **Table S6.** Clinical characteristics of non-COVID-19 patients

|  | **Non-COVID-19 patients (n=27)** |
| --- | --- |
| Pulmonary ARDS | 14 (51.9%) |
| Extrapulmonar ARDS | 13 (48.1%) |
| **Underlying Conditions*, n (%)** |  |
| Bronchopneumonia | 14 (51.9%) |
| Pneumocystosis | 4 (14.8%) |
| Cytomegalovirus | 4 (14.8%) |
| Respiratory syncytial virus | 1 (3.7%) |
| Aspergillosis | 1 (3.7%) |
| Influenza H1N1 | 1 (3.7%) |
| Liver diseases | 11 (40.7%) |
| Extrapulmonary sepsis | 9 (33.3%) |
| Gastrointestinal diseases | 7 (25.9%) |
| Gastrointestinal bleeding | 5 (18.5%) |
| Cardiovascular diseases | 5 (18.5%) |
| Neurologic diseases | 5 (18.5%) |
| Renal diseases | 4 (14.8%) |

*Some patients may have more than one underlying condition.

## **Table S7.** Demographic and clinical characteristics of COVID-19 and non-COVID-19 subgroups divided according to ventilation duration.

|  | **COVID-19**  **L7**  **(n=10)** | **COVID-19**  **H7**  **(n=18)** | **non-COVID-19**  **L7**  **(n=17)** | **non-COVID-19**  **H7**  **(n=10)** | **p-value** |
| --- | --- | --- | --- | --- | --- |
| **Age in years,** mean ± SD | 56.8 ± 19.5 | 57 ±15.1 | 47.1 ± 15.8 | 40.5 ± 12.2 | 0.051 |
| **Body Mass Index (Kg/m^2^),** mean ± SD | 25.8 ± 8.1 | 27.1 ± 7.8 | 24.2 ± 2.5 | 24.2 ± 2.5 | 0.547 |
| **Sex,** n (%) |  |  |  |  | 0.150 |
| Male | 8 (80%) | 9 (50%) | 6 (35.3%) | 6 (60%) |  |
| Female | 2 (20%) | 9 (50%) | 11 (64.7%) | 4 (40%) |  |
| **Race (self-declared),** n (%) |  |  |  |  | 0.346 |
| White | 9 (90%) | 15 (83.3%) | 12 (70.6%) | 6 (60%) |  |
| Afro-descendent | 1 (10%) | 3 (16.7%) | 5 (29.4%) | 4 (40%) |  |
| **Smoking,** n (%) |  |  |  |  | 0.322 |
| Yes | 0 | 2 (11.1%) | 4 (23.5%) | 0 |  |
| Former | 2 (20%) | 5 (27.8%) | 3 (17.6%) | 1 (10%) |  |
| **Time from symptom onset to hospitalization in days**, mean ± SD | 5.9 ± 3.7 | 5.39 ± 3.5 | - | - | 0.646* |
| **Time from symptom onset to death in days**, mean ± SD | 11.5 ± 7.6 | 21.7 ± 7.1 | - | - | 0.021* |
| **Period of hospitalization in days**, mean ± SD | 5.6 ± 4.6 | 16.3 ± 5.8 | 16.6 ± 16.7 | 20.9 ± 5.8^A^ | 0.001 |
| **Mechanical ventilation duration in days**, mean ± SD | 2.4 ± 1.9 | 13 ± 4.8^BC^ | 2.2 ± 1.6 | 15.4 ± 7^BC^ | <0.0001 |
| **PaO_2_/FiO_2_ ratio**, mean ± SD | 163 ± 74.8 | 151.3 ± 106.6 | 135.1 ± 69.3 | 212.4 ± 156.9 | 0.755 |

L7: up to 6 days of mechanical ventilation. H7: 7 days or more of mechanical ventilation. * Comparison between the groups COVID-19 L7 and COVID 19 H7. A: p=0.011 compared to COVID-19 L7. B: p<0.0001 compared to COVID-19 L7. C: p<0.0001 compared to non COVID-19 L7.

## **Table S8.** Demographic and clinical characteristics of male and female patients of COVID-19 and non-COVID-19 patients.

|  | **COVID-19**  **Female**  **(n=11)** | **COVID-19**  **Male**  **(n=17)** | **p-value** | **non-COVID-19**  **Female**  **(n=15)** | **non-COVID-19**  **Male**  **(n=12)** | **p-value** |
| --- | --- | --- | --- | --- | --- | --- |
| **Age in years,** mean ± SD | 56.8 ± 14.6 | 57 ±17.9 | 0.978 | 48.3 ± 16.1 | 40.1 ± 11.72 | 0.154 |
| **Body Mass Index (Kg/m^2^),** mean ± SD | 24.7 ± 3.5 | 27.8 ± 9.6 | 0.309 | 25.7 ± 3.5 | 23.7 ± 2.1 | 0.256 |
| **Race (self-declared),** n (%) |  |  | 0.527 |  |  | 0.660 |
| White | 10 (90.9%) | 14 (82.4%) |  | 10 (66.6%) | 8 (66.6%) |  |
| Afro-descendent | 1 (9.1%) | 3 (17.6%) |  | 5 (33.3%) | 4 (33.3%) |  |
| **Smoking,** n (%) |  |  | 0.320 |  |  | 0.367 |
| Yes | 0 | 2 (11.8%) |  | 2 (13.3%) | 2 (16.6%) |  |
| Former | 4 (36.4%) | 3 (17.6%) |  | 1 (6.6%) | 3 (25%) |  |
| **Time from symptom onset to hospitalization in days**, mean ± SD | 6.4 ±4.7 | 5 ± 2.5 | 0.360 | - | - | - |
| **Time from symptom onset to death in days**, mean ± SD | 20.2 ± 7.2 | 16.7 ± 9.5 | 0.312 | - | - | - |
| **Period of hospitalization in days**, mean ± SD | 13.7 ± 5.6 | 11.7 ± 8.5 | 0.496 | 15.5 ± 10.9 | 21.6 ± 16.4 | 0.300 |
| **Mechanical ventilation duration in days**, mean ± SD | 10.5 ± 5.3 | 8.3 ± 7.2 | 0.394 | 6.4 ± 7.5 | 8 ± 8.4 | 0.719 |
| **PaO_2_/FiO_2_ ratio**, mean ± SD | 142.2 ± 54.2 | 164.2 ± 119.2 | 0.717 | 145 ± 95.5 | 187.1 ± 133.7 | 0.399 |

## **Table S9.** Concentration levels of the cytokines/chemokines of the COVID-19 cases.

| Cytokine | n | pg/mL |
| --- | --- | --- |
| TGF-beta 1 | 24 | 85003.4 ± 71184.0 |
| TGF-beta 2 | 24 | 37052.4 ± 36286.5 |
| TGF-beta 3 | 24 | 27196.6 ± 30062.6 |
| MMP-1 | 24 | 2127 ± 3735.7 |
| MMP-2 | 24 | 24441.5 ± 24826.3 |
| MMP-9 | 24 | 155311.1 ± 232208.7 |
| MMP-10 | 24 | 730.7 ± 848.6 |
| TIMP 2 | 24 | 168654.5 ± 224049.3 |
| IFN- alpha 2 | 15 | 640.6 ± 859.8 |
| IFN-gamma | 24 | 4418.5 ± 3418.4 |
| IL-1 beta | 24 | 7573.3 ± 8770.2 |
| IL-1 Ra | 15 | 92748 ± 108990.2 |
| IL-4 | 24 | 8815.1 ± 14218.6 |
| IL-6 | 24 | 31817.8 ± 43442.1 |
| IL-8 | 24 | 75622.5 ± 110218.8 |
| IL-10 | 24 | 4241.4 ± 4226.3 |
| IL-17A | 15 | 152.6 ± 125.2 |
| IP-10 | 24 | 57392.2 ± 138718.2 |
| MCP1 | 15 | 103445.5 ± 174355.2 |
| MDC/CCL22 | 24 | 7455.6 ± 5911.7 |
| MIG/CXCL9 | 24 | 24016.3 ± 27821.1 |
| MIP-1/CCL15 | 24 | 21235.8 ± 21185.3 |
| MIP-3/CCL20 | 24 | 10010.1 ± 20130.7 |
| TARC/CCL17 | 24 | 2466.1 ± 2947.7 |
| TNF-alpha | 24 | 3296.9 ± 2669.4 |

Data expressed in mean ± standard deviation.

## **Table S10.** DEGs expression and enriched GO terms in the DEGs that are exclusive to the COVID-19 L7 and control samples comparison. BP = biological process, MF = Molecular Function, CC = Cellular Component.

## **Table S11.** DEGs expression and enriched GO terms in the DEGs that are exclusive to the COVID-19 H7 and control samples comparison. BP = biological process, MF = Molecular Function, CC = Cellular Component.

## **Table S12.** DEGs expression and enriched GO terms in the DEGs that are exclusive to the COVID-19 L7 and COVID-19 H7 comparison. BP = biological process, MF = Molecular Function, CC = Cellular Component.

# **References**

1. Erjefält JS, de Souza XCN, Jönsson J, Cozzolino O, Dantas KC, Clausson CM, et al. Diffuse alveolar damage patterns reflect the immunological and molecular heterogeneity in fatal COVID-19. EBioMedicine 2022; 83:104229. doi: 10.1016/j.ebiom.2022.104229.
2. Love MI, Huber W, Anders S. Moderated estimation of fold change and dispersion for RNA-seq data with DESeq2. Genome Biol 2014; 15(12):550. doi: 10.1186/s13059-014-0550-8.
3. Wu T, Hu E, Xu S, Chen M, Guo P, Dai Z, et al. ClusterProfiler 4.0: A universal enrichment tool for interpreting omics data. Innovation (Camb) 2021; 2(3):100141. doi: 10.1016/j.xinn.2021.100141.
4. Yu G, Wang LG, Han Y, He QY. ClusterProfiler: an R package for comparing biological themes among gene clusters. OMICS 2012; 16(5):284-287. doi: 10.1089/omi.2011.0118.
5. Benjamini Y, Hochberg Y. Controlling the False Discovery Rate: A Practical and Powerful Approach to Multiple Testing. J R Stat Soc Series B Stat Methodol 1995; 57:289–300. doi: 10.1111/j.2517-6161.1995.tb02031.x.
